# Evidence-informed health policy 2 – Survey of organizations that support the use of research evidence

**DOI:** 10.1186/1748-5908-3-54

**Published:** 2008-12-17

**Authors:** John N Lavis, Elizabeth J Paulsen, Andrew D Oxman, Ray Moynihan

**Affiliations:** 1 Centre for Health Economics and Policy Analysis, Department of Clinical Epidemiology and Biostatistics, McMaster University, 1200 Main St West, HSC-2D3, Hamilton, ON L8N 3Z5, Canada; 2 Department of Political Science, McMaster University, 1200 Main St West, HSC-2D3, Hamilton, ON L8N 3Z5, Canada; 3 Norwegian Knowledge Centre for the Health Services, Pb 7004, St Olavs plass, Oslo N-0130, Norway; 4 School of Medicine and Public Health, Faculty of Health, the University of Newcastle, Medical Sciences Building – Level 6, Callaghan, NSW 2308, Australia

## Abstract

**Background:**

Previous surveys of organizations that support the development of evidence-informed health policies have focused on organizations that produce clinical practice guidelines (CPGs) or undertake health technology assessments (HTAs). Only rarely have surveys focused at least in part on units that directly support the use of research evidence in developing health policy on an international, national, and state or provincial level (*i.e*., government support units, or GSUs) that are in some way successful or innovative or that support the use of research evidence in low- and middle-income countries (LMICs).

**Methods:**

We drew on many people and organizations around the world, including our project reference group, to generate a list of organizations to survey. We modified a questionnaire that had been developed originally by the Appraisal of Guidelines, Research and Evaluation in Europe (AGREE) collaboration and adapted one version of the questionnaire for organizations producing CPGs and HTAs, and another for GSUs. We sent the questionnaire by email to 176 organizations and followed up periodically with non-responders by email and telephone.

**Results:**

We received completed questionnaires from 152 (86%) organizations. More than one-half of the organizations (and particularly HTA agencies) reported that examples from other countries were helpful in establishing their organization. A higher proportion of GSUs than CPG- or HTA-producing organizations involved target users in the selection of topics or the services undertaken. Most organizations have few (five or fewer) full-time equivalent (FTE) staff. More than four-fifths of organizations reported providing panels with or using systematic reviews. GSUs tended to use a wide variety of explicit valuation processes for the research evidence, but none with the frequency that organizations producing CPGs, HTAs, or both prioritized evidence by its quality. Between one-half and two-thirds of organizations do not collect data systematically about uptake, and roughly the same proportions do not systematically evaluate their usefulness or impact in other ways.

**Conclusion:**

The findings from our survey, the most broadly based of its kind, both extend or clarify the applicability of the messages arising from previous surveys and related documentary analyses, such as how the 'principles of evidence-based medicine dominate current guideline programs' and the importance of collaborating with other organizations. The survey also provides a description of the history, structure, processes, outputs, and perceived strengths and weaknesses of existing organizations from which those establishing or leading similar organizations can draw.

## Background

Organizations that support the use of research evidence in developing health policy can do so in many ways. Some produce clinical practice guidelines (CPGs) or more generally guidance for clinicians and public health practitioners. Others undertake health technology assessments (HTAs) with a focus on informing managerial and policy decisions about purchasing, coverage, or reimbursement. Still others directly support the use of research evidence in developing health policy on an international, national, and state or provincial level (hereafter called government support units, or GSUs). As we argued in the introductory article in the series, a review of the experiences of such organizations, especially those based in low- and middle-income countries (LMICs) and that are in some way successful or innovative, can reduce the need to 'reinvent the wheel' and inform decisions about how best to organize support for evidence-informed health policy development processes, particularly in LMICs [1].

We focus here on describing the methods and findings from the first phase of a three-phase, multi-method study (Table 1) [2]. In this phase we surveyed a senior staff member (the director or his or her nominee) of CPG-producing organizations, HTA agencies, and GSUs about their history, structure, processes, outputs, and perceived strengths and weaknesses. Previous surveys of organizations that support the development of evidence-informed health policies have focused on organizations that produce CPGs [3-10], or undertake HTAs [11-14]. Only rarely have surveys focused at least in part on GSUs [15], or on organizations that are in some way successful or innovative [9], and to our knowledge surveys have never focused at least in part on organizations that support the use of research evidence in LMICs. In the following two articles in the series, we provide more detail about the methods and findings from the interview and case descriptions phases of the study [16,17].

**Table 1:** Overview of the four-article series

[1]	Synthesis of findings from the three-phase, multi-method study
**This article**	**Survey of a senior staff member (the director or his or her nominee) of clinical practice guideline-producing organizations, HTA agencies, and government support units**

[16]	Interview with the senior staff member of a purposively sampled sub-group of these three types of organizations, with an emphasis on those organizations that were particularly successful or innovative

[17]	Case descriptions (based on site visits) of one or more organizations supporting the use of research evidence from among the cases described in the interviews and (once) other cases with which we were familiar, again with an emphasis on those organizations that were particularly successful or innovative

## Methods

We drew on many people and organizations around the world, including our project reference group, to generate a list of organizations to survey [2]. We modified a questionnaire that had been developed originally by the Appraisal of Guidelines for Research and Evaluation (AGREE) collaboration, adapted one version of the questionnaire for organizations producing CPGs and HTAs and another for GSUs, piloted both versions of the questionnaire, and made a small number of final modifications to both versions of the questionnaire. We sent the questionnaire by email to 176 organizations and followed up periodically with non-responders by email and telephone.

### Study population

Eligible CPG-producing organizations, HTA agencies, and GSUs had to perform at least one of the following functions (or a closely related function): 1) produce systematic reviews, HTAs, or other types of syntheses of research evidence in response to requests from decision-makers (*i.e*., clinicians, health system managers, and public policymakers); 2) identify and contextualise research evidence in response to requests from decision-makers; and/or 3) plan, commission, or carry out evaluations of health policies in response to requests from decision-makers. The GSUs could include units located within a health system, government or international organization, units hosted within a university or other research-intensive organization, and independent units with a mandate to directly support evidence-informed health policy (including health care policy, public health policy, and healthy public policy). We excluded organizations that receive core funding from industry (*e.g*., pharmaceutical companies) or that only produce or provide health or healthcare utilization data.

While we included all eligible organizations from LMICs, for high-income countries we included: 1) established CPG-producing organizations that are members of the Guidelines International Network (GIN) and select other organizations that are known to produce CPGs in particularly innovative or successful ways; 2) established HTA agencies that are members of the International Network of Agencies for Health Technology Assessment (INAHTA) and select other HTA agencies that are known to produce HTAs in particularly innovative or successful ways; and 3) any units that directly support the use of research evidence in developing health policy. We drew on members of both formal and informal international networks to identify particularly innovative or successful CPG-producing organizations and HTA agencies and to identify GSUs. The formal networks included the Appraisal of Guidelines for Research and Evaluation (AGREE) collaboration, the Cochrane Collaboration, GIN, GRADE Working Group, International Clinical Epidemiology Network (INCLEN) Knowledge Management Program, and INAHTA. The informal networks included our project reference group, staff at WHO headquarters and regional offices, and personal networks.

### Survey development and administration

We drew on a questionnaire developed and used by the AGREE collaboration [9], and we modified questions as necessary given our focus on LMICs. The questions covered seven domains: 1) organization; 2) why and how the organization was established; 3) focus; 4) people involved; 5) methodology employed; 6) products and implementation; and 7) evaluation and update procedures. We also included a final group of additional questions. About two-fifths of the questions were open-ended. Two of the questions were changed for the version of the questionnaire administered to GSUs; this questionnaire had 48 questions instead of 49. We piloted the questionnaire with three organizations in each category (and received responses from five organizations). See 'Additional file 1: Questionnaire – CPG & HTA' for the questionnaire for units producing CPGs or HTAs, and see 'Additional file 2: Questionnaire – GSU' for the questionnaire for units supporting health policy.

We sent the questionnaire by email to the director (or another appropriate person) of each eligible organization with three options for responding: by answering questions in the body of our email message and returning it; by answering questions in a Word version of our questionnaire attached to our e-mail message and returning it; or by printing a PDF version of our questionnaire, completing it by hand, and mailing it. We sent three reminders if we did not receive a response (at roughly 2, 8 and 10 weeks after the original contact for most organization and at roughly 1, 2.5 and 4 weeks for the organizations for which we had difficulty tracking down contact information), each time offering to re-send the questionnaire upon request. We used additional mechanisms to increase the response rate, including an endorsement letter and personal contacts [18].

### Data management and analysis

Quantitative data were entered manually and summarized using simple descriptive statistics. Written comments were grouped by question, and one member of the team (RM) identified themes using a constant comparative method of analysis. The findings were then independently reviewed by two members of the research team (AO and JL).

The principal investigator for the overall project (AO), who is based in Norway, confirmed that, in accordance with the country's act on ethics and integrity in research, this study did not require ethics approval from one of the country's four regional committees for medical and health research ethics. In keeping with usual conventions in survey research, we took the voluntary completion and return of the survey as indicating consent. We did not mention either treating participants' responses as confidential data or safe-guarding participants' anonymity in our initial request to participate in the study or in the questionnaire itself. Nevertheless, we present only aggregated data and take care to ensure that no individuals or organizations can be identified. We shared a report on our findings with participants and none of them requested any changes to how we present the data.

## Results

We sent 176 questionnaires, and 152 (86%) completed questionnaires were returned. Ninety-five organizations produce CPGs, HTAs, or both and 57 units support government policymaking (*i.e*., are what we call GSUs) (Table 2). Twenty-nine organizations were identified through the GIN membership list, 26 through INAHTA, and 82 through personal contacts, including 49 of the 57 GSUs.

**Table 2:** Description of the units

Characteristics	Organizations producing CPGs (n = 31)	Organizations producing HTAs (n = 19)	Organizations producing CPGs and HTAs (n = 45)	Organizations supporting government policymaking (n = 57)	All (n = 152)
**Source from which units identified**					

GIN	13	0	13	3	29

INAHTA	1	17	7	1	26

INCLEN	5	0	5	4	14

Personal contacts	11	2	20	49	82

Other	1	0	0	0	1

**Economic classification of the countries in which they units are based**					

Low-income	0	0	2	6	8

Lower-middle income	8	1	9	18	36

Upper-middle income	3	0	5	11	19

High income	20	18	27	20	85

**Region in which they units are based**					

Africa	1	0	1	5	7

Asia	4	1	9	18	32

Australia and New Zealand	1	2	4	2	9

Eastern Europe	0	0	1	0	1

Western Europe	13	12	15	9	49

Latin America and the Caribbean	6	0	6	9	21

Middle East	0	1	0	3	4

North America	6	3	8	8	25

International	0	0	1	2	3

Although we intentionally sought out organizations in LMIC, 56% (n = 85) were from high-income countries, 13% (n = 19) from upper middle-income countries, 24% (n = 36) from lower middle-income countries and 5% (n = 8) from low-income countries. Over one-half the organizations (54%) that produced CPGs and HTAs were identified through GIN and INAHTA (51/95), and 68% (n = 65) were from high-income countries compared to 35% (20/57) of GSUs. Although we aimed to identify organizations throughout the world, the included organizations were not spread evenly across different regions. Sixty-seven percent (64/95) of the organizations that produce CPGs and HTAs were located in Western Europe (n = 40), North America (n = 17), Australia and New Zealand (n = 7), compared with 33% of GSUs (19/57). We identified few organizations in Eastern Europe (n = 1), India (n = 2), the Middle East (n = 3) or China (n = 4) that met our inclusion criteria, and only three international organizations were included.

### Quantitative results

#### Organization and establishment

A high proportion of organizations that produce CPGs, HTAs, or both also support government policymaking in other ways, whereas the reverse (GSUs producing CPGs or HTAs) was much less common (Table 3). Among the array of services undertaken in response to requests from public policymakers, GSUs are most likely to convene expert meetings to discuss available research (82%) and undertake short-term research projects (79%). Organizations that produce CPGs were often based in professional associations (45%) whereas organizations that produce HTAs, or both CPGs and HTAs, were often based in government agencies (63% and 49%, respectively). GSUs were also often based in academic institutions (37%) and government agencies (39%). HTA agencies were particularly likely to receive funding from government sources (95%), whereas the other types of organizations did not have such a commonly shared revenue source. More than one-half of the organizations (and particularly HTA agencies) reported that examples from other countries were helpful in establishing their organization.

**Table 3:** Organization and establishment

Characteristics	Organizations producing CPGs (n = 31)	Organizations producing HTAs (n = 19)	Organizations producing CPGs and HTAs (n = 45)	Organizations supporting government policymaking (n = 57)
**Type of product produced**				

Reported also providing direct support to policymakers for developing health policy	17 (55%)	16 (85%)	35 (78%)	-
Reported also producing clinical practice guidelines	-	-	-	17 (30%)
Reported also producing HTAs	-	-	-	11 (19%)

Types of services undertaken in response to requests from public policymakers*				

Identify primary research	-	-	-	36 (63%)
Identify systematic reviews of research	-	-	-	31 (54%)
Identify clinical practice guidelines, HTAs or other prescriptive research-based documents	-	-	-	24 (42%)
Undertake short-term research projects	-	-	-	45 (79%)
Undertake systematic reviews of research	-	-	-	38 (67%)
Commission systematic reviews of research	-	-	-	17 (30%)
Either undertake systematic reviews of research or commission systematic reviews of research	-	-	-	41 (72%)
Convene expert meetings to discuss available research	-	-	-	47 (82%)
Other	-	-	-	18 (32%)

**Type of organization**				

Academic institution	7 (23%)	7 (37%)	11 (24%)	21 (37%)
Disease-specific association	1 (3%)	0 (0%)	1 (2%)	0 (0%)
Professional association	14 (45%)	2 (11%)	4 (9%)	3 (5%)
Government agency	9 (29%)	12 (63%)	22 (49%)	22 (39%)
International agency	0 (0%)	0 (0%)	3 (7%)	7 (12%)
Other	8 (26%)	2 (11%)	7 (16%)	18 (32%)

Source of funding*				

Biomedical or other for-profit company	7 (23%)	1 (5%)	6 (13%)	6 (11%)
Government	17 (55%)	18 (95%)	38 (84%)	45 (79%)
Other	15 (48%)	3 (16%)	20 (44%)	38 (67%)

**Examples from other countries helpful in establishing the organization**				

Yes	18 (58%)	14 (74%)	24 (53%)	30 (53%)
No	8 (26%)	0 (0%)	15 (33%)	17 (30%)
Not reported	4 (13%)	4 (21%)	6 (13%)	7 (12%)

*More than one answer was possible for the question

#### Age, budget and production profile

The organizations' ages, budgets, and production profiles varied dramatically (Table 4). The median age was 7 to 10 years depending on the type of organization; however, one organization had just begun directly supporting the use of research evidence in developing health policy and another had a 94-year history. The median annual budget was lowest for CPG-producing organizations and highest for HTA-producing organizations. The median number of CPGs or HTAs produced per year ranged from three to seven, and the median time spent to produce a CPG or HTA ranged from 10 to 15 months.

**Table 4:** Age, budget and production profile

Characteristics	Organizations producing CPGs (n = 31)			Organizations producing HTAs (n = 19)			Organizations producing CPGs and HTAs (n = 45)			Organizations supporting government policymaking (n = 57)		
	
	n	Median	Range	n	Median	Range	n	Median	Range	n	Median	Range
Median years since began production/service	28	9	2 to 27	18	8	3 to 20	44	7	1 to 27	55	10	0 to 94

Median annual budget (in US dollars)	26	368,275	500 to 15,000,000	16	875,000	125,000 to 21,600,000	38	700,000	5,000 to 40,000,000	41	692,000	1,200 to 51,000,000

Median number of CPGs or HTAs produced per year	31	3	0.5 to 500	17	7	2 to 45	42	7	1 to 300	-	-	-

Median time for production of a CPG or HTA (in months)	31	15	0.3 to 33	17	12	4 to 36	41	10	0.3 to 30	-	-	-

#### Focus

Organizations producing CPGs were more often focused on health care (65–84%) than on public health (45%) or healthy public policy (26%), whereas GSUs were more focused on public health (88%) and to a lesser extent on primary healthcare (72%) and healthy public policy (67%) (Table 5). A high proportion of GSUs provided service on many facets of policy issues: characterizing problems (74%), identifying potential solutions (82%), fitting solutions into health systems (75%), and bringing about change in health systems (88%). Organizations producing CPGs were more focused on physicians (100%) and to a lesser extent other types of healthcare providers (77%) as their target users, whereas HTA agencies were more focused on health system managers (95%) and public policymakers (100%). GSUs were most focused on public policymakers in health departments, followed by public policymakers in central agencies (77%), stakeholders (79%), and public policymakers in other departments (63%). A higher proportion of GSUs involved target users in the selection of topics or the services undertaken than CPG- or HTA-producing organizations.

**Table 5:** Focus

Characteristics	Organizations producing CPGs (n = 31)	Organizations producing HTAs (n = 19)	Organizations producing CPGs and HTAs (n = 45)	Organizations supporting government policymaking (n = 57)
Domains from which topics are selected*				

Primary healthcare	26 (84%)	18 (95%)	38 (84%)	41 (72%)

Secondary healthcare	25 (81%)	18 (95%)	33 (73%)	29 (51%)

Tertiary healthcare	20 (65%)	18 (95%)	32 (71%)	26 (46%)

Public health (*i.e*., health is the objective)	14 (45%)	15 (79%)	33 (73%)	50 (88%)

Health public policy	8 (26%)	9 (47%)	21 (47%)	38 (67%)

Domains in which service is provided*				

Characterizing the problem	-	-	-	42 (74%)

Identifying potential solutions to health problems	-	-	-	47 (82%)

Fitting solutions into health systems (*i.e*., governance, financial and delivery arrangements)	-	-	-	43 (75%)

Bringing about change in health systems	-	-	-	50 (88%)

Target users*				

Patients/public	18 (58%)	13 (68%)	32 (71%)	-

Physicians	31 (100%)	17 (89%)	43 (96%)	-

Other types of healthcare providers	24 (77%)	15 (79%)	35 (78%)	-

Healthcare managers	18 (58%)	18 (95%)	36 (80%)	-

Public policymakers	18 (58%)	19 (100%)	37 (82%)	-

Public policymakers in health departments	-	-	-	50 (88%)

Public policymakers in other departments	-	-	-	36 (63%)

Public policymakers in central agencies (*e.g*., executive branch)	-	-	-	44 (77%)

Stakeholders	-	-	-	45 (79%)

**Involvement of target users in selection of topics or services undertaken**				

By participation in priority-setting group or working groups	18 (58%)	13 (68%)	28 (62%)	50 (88%)

By survey of views/preferences	14 (45%)	6 (32%)	20 (44%)	35 (61%)

By review of draft list of priority topics or draft reports	15 (48%)	5 (26%)	21 (27%)	43 (75%)

No	6 (19%)	2 (11%)	7 (16%)	0 (0%)

Not reported	1 (3%)	1 (5%)	1 (2%)	3 (5%)

*More than one answer was possible for the question

#### People involved in producing a product or delivering a service

Most organizations have a small number of full-time equivalent (FTE) staff (Table 6). For example, more than one-half of organizations producing CPGs, HTAs, or both have between one and five FTE staff. More than one-half of all organizations always involved an expert in information/library science, and more than two-thirds of CPG- and HTA-producing organizations always involved an expert in clinical epidemiology. More than one-half of all HTA agencies also always involved a health economist and (only if necessary) involved experts in biostatistics, other types of social scientists, and a consumer representative. More than two-thirds of organizations producing CPGs or both CPGs and HTAs involve target users by inviting them to participate in the development group or to review the draft product. A higher proportion of GSUs than other types of organizations involve consumers in product development or service delivery. For example, 44% of GSUs invite consumers to participate in the development group and 54% survey their views/preferences. More than two thirds of organizations producing CPGs consider geographic balance in expert or target user selection, but a lower proportion of other types of organizations use this criterion.

**Table 6:** People involved in producing a product or delivering a service

Characteristics	Organizations producing CPGs (n = 31)	Organizations producing HTAs (n = 19)	Organizations producing CPGs and HTAs (n = 45)	Organizations supporting government policymaking (n = 57)
**Average number of members in a CPG or HTA development panel**				

1–5 full time equivalents (FTE)	17 (55%)	11 (58%)	23 (51%)	-

6–10 FTE	9 (29%)	5 (26%)	11 (24%)	-

11–15 FTE	2 (6%)	0 (0%)	5 (11%)	-

16–20 FTE	1 (3%)	1 (5%)	1 (2%)	-

> 20 FTE	2 (6%)	1 (5%)	3(7%)	-

Not reported	1 (3%)	1 (5%)	1 (2%)	-

**Average number of staff involved in service delivery**				

< 0.5 full time equivalents (FTE)	-	-	-	8 (14%)

0.5 – 1.9 FTE	-	-	-	6 (11%)

> 2 FTE	-	-	-	37 (65%)

Types of experts/stakeholders who were always involved*				

Information/library science	19 (61%)	16 (84%)	27 (60%)	29 (51%)

Clinical epidemiology	21 (68%)	14 (74%)	32 (71%)	24 (42%)

Biostatistics	3 (10%)	6 (32%)	17 (38%)	23 (40%)

Health economics	5 (16%)	10 (53%)	17 (38%)	24 (42%)

Other types of social scientists	4 (13%)	4 (21%)	12 (27%)	23 (40%)

Knowledge transfer/communications	10 (32%)	8 (42%)	21 (47%)	24 (42%)

Consumer	9 (29%)	3 (16%)	17 (38%)	15 (26%)

Other	7 (23%)	9 (47%)	13 (29%)	15 (26%)

Types of experts/stakeholders who were involved only if necessary*				

Information/library science	10 (32%)	2 (11%)	13 (29%)	22 (39%)

Clinical epidemiology	8 (26%)	4 (21%)	11 (24%)	22 (39%)

Biostatistics	16 (52%)	12 (63%)	19 (42%)	26 (46%)

Health economics	15 (48%)	9 (47%)	23 (51%)	26 (46%)

Other types of social scientists	14 (45%)	15 (79%)	26 (58%)	24 (42%)

Knowledge transfer/communications	8 (26%)	8 (42%)	9 (20%)	23 (40%)

Consumer	12 (39%)	11 (58%)	11 (24%)	24 (42%)

Other	2 (6%)	4 (21%)	3 (7%)	5 (9%)

**Involvement of target users in product development or service delivery**				

By participation in development/delivery group	25 (81%)	11 (58%)	33 (73%)	-

By survey of views/preferences	9 (29%)	2 (11%)	18 (40%)	-

By review of draft product or service model	22 (71%)	9 (47%)	32 (71%)	-

No	0 (0%)	3 (16%)	3 (7%)	

Not reported	0 (0%)	2 (11%)	1 (2%)	-

**Involvement of consumers (patients or general public) in product development or service delivery**				

By participation in development group	12 (39%)	3 (16%)	16 (36%)	25 (44%)

By survey of views/preferences	9 (29%)	2 (11%)	14 (31%)	31 (54%)

By review of draft guideline (or HTA)	14 (45%)	5 (26%)	23 (51%)	17 (30%)

No	10 (32%)	9 (47%)	13 (29%)	15 (26%)

Not reported	1 (3%)	2 (11%)	1 (2%)	1 (2%)

Criteria used in expert and/or target user selection*				

Geographic balance	21 (68%)	7 (37%)	18 (40%)	-

Gender balance	8 (26%)	2 (11%)	8 (18%)	-

Other	18 (58%)	11 (58%)	25 (56%)	-

*More than one answer was possible for the question

#### Methods used in producing a product or delivering a service

Organizations draw on a wide variety of types of information (Table 7). More than four-fifths (84 to 100%) of organizations reported providing panels with or using systematic reviews. Organizations producing CPGs, HTAs, or both tended to use an explicit valuation process for the research evidence (89 to 97% prioritized evidence by its quality), but used one less often for outcomes (52 to 61% prioritized outcomes by their importance to those affected), and still less often for groups (0 to 26% prioritized groups by their importance to achieving equity objectives). GSUs tended to use a wide variety of explicit valuation processes, but none with the frequency that organizations producing CPGs, HTAs, or both prioritized evidence by its quality. A higher proportion of organizations producing CPGs, HTAs, or both graded recommendations according to the quality of the evidence and/or the strength of the recommendation than used other methods to formulate recommendations. Roughly one-half of GSUs used each of subjective review, consensus, and grading to formulate recommendations. A higher proportion of organizations producing CPGs, HTAs, or both explicitly assessed the quality of evidence in formulating recommendations than explicitly assessed the trade-offs between benefits and harms, costs or equity. Almost one-half of GSUs explicitly assessed equity in formulating recommendations. A higher proportion of organizations used internal review or external review by experts than other review processes.

**Table 7:** Methods used in producing a product or delivering a service

Characteristics	Organizations producing CPGs (n = 31)	Organizations producing HTAs (n = 19)	Organizations producing CPGs and HTAs (n = 45)	Organizations supporting government policymaking (n = 57)
Types of information provided to the panel or employed*				

Systematic reviews	31 (100%)	16 (84%)	42 (93%)	49 (86%)

Economic evaluations	15 (48%)	15 (79%)	24 (53%)	34 (60%)

Decision analyses	13 (42%)	9 (47%)	23 (51%)	29 (51%)

Existing burden of disease/illness	26 (84%)	17 (89%)	34 (76%)	38 (67%)

Existing practice patterns	21 (68%)	14 (74%)	33 (73%)	33 (58%)

Existing guidelines (or HTAs)	30 (97%)	15 (79%)	41 (91%)	38 (67%)

Resource constraints	11 (35%)	10 (53%)	18 (40%)	26 (46%)

Commissioned research	-	-	-	27 (47%)

Other	7 (23%)	5 (26%)	9 (20%)	10 (18%)

Explicit valuation process used*				

Evidence is prioritized by its quality	30 (97%)	17 (89%)	41 (91%)	40 (70%)

Outcomes are prioritized by their importance to those affected	19 (61%)	10 (53%)	28 (52%)	39 (68%)

Groups are prioritized by their importance to achieving equity objectives	8 (26%)	0 (0%)	11 (24%)	27 (47%)

Methods used to formulate recommendations*				

Subjective review	5 (16%)	5 (26%)	12 (27%)	26 (46%)

Informal consensus	10 (32%)	9 (47%)	11 (24%)	28 (49%)

Formal consensus (*e.g*., nominal group or Delphi techniques)	18 (58%)	3 (16%)	19 (42%)	29 (51%)

Graded according to the quality of the evidence and/or the strength of the recommendation (using an explicit rating scheme)	26 (84%)	12 (63%)	34 (76%)	31 (54%)

Explicit assessments used in formulating recommendations*				

Quality of evidence	29 (94%)	16 (84%)	43 (96%)	41 (72%)

Trade-offs between benefits and harms	20 (65%)	9 (47%)	35 (78%)	35 (61%)

Costs	14 (45%)	12 (63%)	30 (67%)	31 (54%)

Equity	9 (29%)	6 (32%)	21 (47%)	27 (47%)

Review processes used*				

Clinical or policy validation (*e.g*., pilot testing, trial implementation period)	9 (29%)	3 (16%)	11 (24%)	24 (42%)

Comparison with products or input from other groups	15 (48%)	5 (26%)	27 (60%)	19 (33%)

Internal review	26 (84%)	17 (89%)	38 (84%)	41 (72%)

External review by experts	24 (77%)	16 (84%)	42 (93%)	43 (75%)

External review by target users	18 (58%)	5 (26%)	21 (47%)	23 (40%)

*More than one answer was possible for the question

#### Products and implementation

All or almost all organizations producing CPGs, HTAs, or both produced a full version of their final product with references, whereas only HTA agencies uniformly produced both the full version and an executive summary (Table 8). Less than one-half of all organizations provided a summary of take-home messages as part of their products. More than two-thirds of organizations producing CPGs, HTAs, or both posted to a website accessed by target users, and more than two thirds of organizations producing HTAs or both CPGs and HTAs mailed or e-mailed products to target users. Only 14% of GSUs submitted products to any form of clearinghouse. More than one-half of organizations were involved in different strategies to develop the capacity of target users to acquire, assess, and use their products or services. Almost two-thirds of GSUs involved target users in an implementation group, whereas lower proportions of other types of organizations involved target users in implementation through this or another approach.

**Table 8:** Products and implementation

Characteristics	Organizations producing CPGs (n = 31)	Organizations producing HTAs (n = 19)	Organizations producing CPGs and HTAs (n = 45)	Organizations supporting government policymaking (n = 57)
Versions produced*				

Full version with notes/references	31 (100%)	19 (100%)	44 (98%)	46 (81%)

Executive summary	20 (65%)	19 (100%)	32 (71%)	40 (70%)

Summary of take-home messages	14 (45%)	5 (26%)	20 (44%)	25 (44%)

Separate summaries/versions for different target users	10 (32%)	5 (26%)	21 (47%)	24 (42%)

Tools for application (*e.g*., algorithms, flow charts)	18 (58%)	2 (11%)	26 (58%)	28 (49%)

Produce at least one of the above 4 versions	27 (87%)	19 (100%)	37 (82%)	49 (86%)

Dissemination/implementation strategies used*				

Send versions of products to the media	21 (68%)	10 (53%)	27 (60%)	32 (56%)

Mail or e-mail products to target users	19 (61%)	15 (79%)	33 (73%)	38 (67%)

Produce a CD-ROM and distribute it to target users	6 (19%)	2 (11%)	17 (38%)	20 (35%)

Post to a website accessed by target users	27 (87%)	13 (68%)	32 (71%)	42 (74%)

Submit to a clearinghouse	16 (52%)	11 (58%)	17 (38%)	8 (14%)

Other	20 (65%)	9 (47%)	18 (40%)	20 (35%)

Other implementation strategies used*				

Patient-mediated interventions	7 (23%)	2 (11%)	11 (24%)	8 (14%)

Provider-mediated interventions	15 (48%)	6 (32%)	18 (40%)	23 (40%)

Organizational interventions	15 (48%)	10 (53%)	18 (40%)	27 (47%)

Strategies used to develop the capacity of target users to acquire, assess and use products they produce or services they deliver*				

Organize training workshops for target users	19 (61%)	12 (63%)	31 (69%)	42 (74%)

Participate in training workshops for target users	17 (55%)	8 (42%)	31 (69%)	36 (63%)

Either organize or participate in training workshops for target users	25 (81%)	12 (63%)	34 (76%)	43 (75%)

Develop a resource document for target users	19 (61%)	10 (53%)	26 (58%)	38 (67%)

Other	5 (16%)	6 (32%)	5 (11%)	5 (9%)

**Involvement of target users in implementation**				

By participation in implementation group	15 (48%)	8 (42%)	26 (58%)	37 (65%)

By survey of views/preferences	9 (29%)	6 (32%)	18 (40%)	28 (49%)

By review of draft implementation strategy	10 (32%)	5 (26%)	14 (31%)	19 (33%)

No	8 (26%)	3 (16%)	5 (11%)	1 (2%)

Not reported	2 (6%)	3 (16%)	9 (20%)	9 (16%)

*More than one answer was possible for the question

#### Evaluation and update procedures

Between one-half and two-thirds of organizations do not collect data systematically about uptake, and roughly the same proportions do not systematically evaluate their usefulness or impact in other ways (Table 9). A little over one-half (52%) of organizations producing CPGs update their products regularly whereas less than one-half (45%) update them irregularly. A higher proportion of other types of organizations update their products and services irregularly (49 to 63%) than regularly (11 to 37%).

**Table 9:** Evaluation and update procedures

Characteristics	Units producing CPGs (n = 31)	Units producing HTAs (n = 19)	Units producing CPGs and HTAs (n = 45)	Units supporting government policymaking (n = 57)
**Collect data systematically about uptake**				

Yes	11 (35%)	6 (32%)	14 (31%)	20 (35%)

No	20 (65%)	13 (68%)	28 (62%)	32 (56%)

Not reported	0 (0%)	0 (0%)	3 (7%)	4 (7%)

**Systematically evaluates usefulness or impact in other ways**				

Yes	10 (32%)	9 (47%)	20 (44%)	23 (40%)

No	21 (68%)	9 (47%)	22 (49%)	29 (51%)

Not reported	0 (0%)	1 (5%)	3 (7%)	5 (9%)

**Updates products and services**				

Updates regularly	16 (52%)	2 (11%)	14 (31%)	21 (37%)

Updates irregularly	14 (45%)	12 (63%)	27 (60%)	28 (49%)

Updates either regularly or irregularly	29 (93%)	13 (68%)	38 (84%)	48 (84%)

Do not update	2 (6%)	5 (26%)	7 (16%)	5 (9%)

Not reported	1 (3%)	1 (5%)	3 (7%)	4 (7%)

### Qualitative results

See additional file 3: Qualitative data for the qualitative data from the survey of organizations that support the use of research evidence

## Discussion

### Principal findings from the survey

A high proportion of organizations that produce CPGs, HTAs, or both also support government policymaking in other ways, whereas the reverse (GSUs producing CPGs or HTAs) was much less common. More than one-half of the organizations (and particularly HTA agencies) reported that examples from other countries were helpful in establishing their organization. The organizations' ages, budgets and production profiles varied dramatically. A higher proportion of GSUs than CPG- or HTA-producing organizations involved target users in the selection of topics or the services undertaken. Most organizations have a small number of FTE staff (*e.g*., five or fewer FTEs for CPG- and HTA-producing organizations). More than one-half of all organizations always involved an expert in information/library science, and more than two-thirds of CPG- and HTA-producing organizations always involved an expert in clinical epidemiology. More than four-fifths of organizations reported providing panels with or using systematic reviews. GSUs tended to use a wide variety of explicit valuation processes for the research evidence, but none with the frequency that organizations producing CPGs, HTAs, or both prioritized evidence by its quality. Less than one-half of all organizations provided a summary of take-home messages as part of their products. Almost two-thirds of GSUs involved target users in an implementation group, whereas lower proportions of other types of organizations involved target users in implementation through this or another approach. Between one-half and two-thirds of organizations do not collect data systematically about uptake, and roughly the same proportions do not systematically evaluate their usefulness or impact in other ways.

For CPG- and HTA-producing organizations, specifically: 1) when they were being established, many conducted a focused review of one particular organization that they then emulated or a broad review of a variety of organizational models; 2) independence is by far the most commonly cited strength in how they are organized and a lack of resources, both financial and human, the most commonly cited weakness; 3) an evidence-based approach is the most commonly cited strength of the methods they use and their methods' time-consuming and labour-intensive nature the most commonly cited weakness; 4) the brand recognition that was perceived to flow from their evidence-based approach, and much less commonly from their strict conflict-of-interest guidelines, is the main strength of their outputs, while the most commonly cited weaknesses were the lack of dissemination and implementation strategies for the outputs and the lack of monitoring and evaluation of impact; 5) the individuals, groups, and organizations who have worked with them or who have benefited from their outputs are their strongest advocates, and the pharmaceutical industry and clinicians who are closely associated with them their strongest critics; and 6) a facilitating role in coordination efforts (in order to avoid duplication) and in local adaptation efforts (in order to enhance local applicability) are their most frequently offered suggestions for WHO and other international agencies and networks.

For GSUs: 1) focusing on the need for secure funding when establishing a GSU was their most commonly offered advice; 2) working within national networks and, more generally, collaborating rather than competing with other bodies, was a commonly cited strength in how these units are organized; 3) government health departments are their strongest advocates; and 4) helping to adapt global evidence to local contexts or at least supporting such processes are their most frequently offered suggestions for WHO and other international agencies and networks. No themes emerged with any consistency among the diverse weaknesses identified in how the units were organized, strengths and weaknesses identified in their methods and outputs, or critics cited.

### Strengths and weaknesses of the survey

The survey has four main strengths: 1) we surveyed the directors of three types of organizations that support evidence-informed policymaking, not just the two types of organizations that are usually studied (*i.e*., we surveyed GSUs as well as CPG- and HTA-producing organizations); 2) we adapted a widely used questionnaire; 3) we drew on a regionally diverse project reference group to ensure that our draft protocol, study population, and questionnaire were fit for purpose; and 4) we achieved a high response rate (86%). The study has two main weaknesses: 1) despite significant efforts to identify organizations in LMICs, just over one-half (54%) of the organizations we surveyed were drawn from high-income countries; and 2) despite efforts to ask questions in neutral ways, many organizations may have been motivated by a desire to tell us what they thought we wanted to hear (*i.e*., there may be a social desirability bias in their responses).

### What the survey adds

The findings from our survey, the most broadly based of its kind, both extend or clarify the applicability (to HTA agencies, GSUs or both) of the messages arising from previous surveys and related documentary analyses and add several new messages. First, our findings concur with the conclusion of the most recent and comprehensive survey of CPG-producing organizations that 'principles of evidence-based medicine dominate current guideline programs' [9], although this was less true of the broad variety of GSUs that we surveyed. Our findings also concur with their conclusion that 'international collaboration should be encouraged to improve guideline methodology and to globalize the collection and analysis of evidence needed for guideline development', which appears equally germane to the HTA agencies and the GSUs we surveyed. Second, our findings concur with the most recent survey of HTA agencies, which focused on the risks to HTA programs (grouped into the categories of formulation of HTA questions, preparation of HTA products, dissemination, and contracting) and the management of those risks, specifically the conclusions related to establishing strong links with policymakers and involving stakeholders in the work, using good methods and being transparent in the work, and being attentive to implementation considerations [14]. Third, our findings concur with the conclusions of several reviews of CPG and HTA documents, particularly the importance of using good methods and being transparent, collaborating with other organizations, and building capacity [19-24]. Our survey also provides a description of the history, structure, processes, outputs, and perceived strengths and weaknesses of GSUs as well as CPG- and HTA-producers, which can be a useful resource for those establishing or leading similar organizations in LMICs.

### Implications for policymakers and for international organizations and networks

Both policymakers and international organizations and networks can play an important facilitating role in coordination efforts (in order to avoid duplication) and in local adaptation efforts (in order to enhance local applicability). They also have an important advocacy role to play in calling for coordination and local adaptation. International organizations and networks can play several additional facilitation roles, particularly in the areas of sharing robust methodologies and where necessary improving existing methodologies (which could include establishing a common framework for evaluations of their impact in order to facilitate cross-organization and cross-jurisdiction learning), collecting and analyzing 'global' research evidence and making it available as an input to 'local' processes, and engaging more organizations based in LMICs and providing training and support for their continued development. Select international organizations, such as the Alliance for Health Policy and Systems Research, may have a particular role to play in sponsoring the development of an international organization of GSUs, which can be difficult to identify, let alone support.

### Implications for future research

The survey should be repeated in a few years on an augmented sample of organizations, including organizations that have self-identified as partners of the Alliance for Health Policy and Systems Research (many of which may be GSUs). Also, as suggested above, there is a need for improving some of the existing methodologies used by the organizations and for establishing a common framework for evaluations of their impact.

## Competing interests

The authors declare that they have no financial competing interests. The study reported herein, which is the first phase of a larger three-phase study, is in turn part of a broader suite of projects undertaken to support the work of the WHO Advisory Committee on Health Research (ACHR). Both JL and AO are members of the ACHR. JL is also President of the ACHR for the Pan American Health Organization (WHO's regional office for the Americas). The Chair of the WHO ACHR, a member of the PAHO ACHR, and several WHO staff members were members of the project reference group and, as such, played an advisory role in study design. Two of these individuals provided feedback on the penultimate draft of the report on which the article is based. The authors had complete independence, however, in all final decisions about study design, in data collection, analysis and interpretation, in writing and revising the article, and in the decision to submit the manuscript for publication.

## Authors' contributions

JL participated in the design of the study, participated in analyzing the qualitative data and deciding how to present the quantitative data, and drafted the article and the report in which it is based. AO conceived of the study, led its design and coordination, participated in analyzing the qualitative data, and contributed to drafting the article. RM participated in the design of the study, led the analysis of the qualitative data, and contributed to drafting the article. EP led the data collection for the study and led the analysis of the quantitative data. All authors read and approved the final manuscript.

## Supplementary Material

Additional file 1 **Questionnaire for units producing clinical practice guidelines or health technology assessments.** This questionnaire is designed to be completed by units or departments that primarily produce clinical practice guidelines (CPGs), and/or produce health technology assessments (HTAs). Click here for file

Additional file 2 **Questionnaire for units supporting health policy.** This questionnaire is designed to be completed by units or departments that primarily provide research evidence and other support for organisations or policymakers developing health policy. Click here for file

Additional file 3 **Qualitative data from the survey of organizations that support the use of research evidence.** Click here for file
