# Supplementary material for: Evidence-informed health policy 2 – Survey of organizations that support the use of research evidence
Source: Implement Sci. 2008 Dec 17;3:54. doi: 10.1186/1748-5908-3-54 (PMC2646748; doi:10.1186/1748-5908-3-54)
Supplement: Additional file 2 — Questionnaire for units supporting health policy. This questionnaire is designed to be completed by units or departments that primarily provide research evidence and other support for organisations or policymakers developing health policy. [file 1748-5908-3-54-S2.doc]

**Evidence-informed health policy:**
A critical review of units that support the use of research evidence in
developing clinical practice guidelines and health policy

**Questionnaire for units supporting health policy**

Thank you for agreeing to complete this questionnaire.

We hope it will produce valuable information how groups around the world use research evidence in developing clinical practise guidelines, health technology assessments and health policy.

Please do not hesitate to contact us at the following email address, if you have any questions about this project: [elizabeth.paulsen@kunnskapssenteret.no](mailto:elizabeth.paulsen@kunnskapssenteret.no).

This questionnaire is designed to be completed by units or departments that primarily provide research evidence and other support for organisations or policymakers developing health policy. If your unit primarily produces clinical practice guidelines (CPGs), and/or produce health technology assessments (HTAs) please email Elizabeth Paulsen at the email address above, and ask for a different questionnaire.

First, can you give the name of your unit or department and provide a very simple description of its work. Please also provide a name, phone number, email and address of someone in your unit that can be contacted for additional information, in case we would like to follow up this survey with a telephone interview or site visit.

| **Name of unit:** |
| --- |
| **Brief description of units work:** |
| **Name of person that can be contacted for additional information:** |
| **Phone:** |
| **Email:** |
| **Address:** |

**ORGANISATION**

1. Type of service undertaken in response to requests from public policymakers *(please put an X next to all that apply)*

____ a. Identify primary research

____ b. Identify systematic reviews of research

____ c. Identify clinical practice guidelines, HTAs or other prescriptive research-based documents

____ d. Undertake short-term research projects

____ e. Undertake systematic reviews of research

____ f. Commission systematic reviews of research

____ g. Convene expert meetings to discuss available research

____ h. Other – *please specify*:

1. Type of organisation

____ a. Academic institution

____ b. Disease specific association

____ c. Professional association (e.g., medical specialty society)

____ d. Biomedical or other for-profit company (e.g., pharmaceutical company)

____ e. Government agency → ___ Local ___ Regional ___ National

____ f. International agency

____ g. Other – *please specify*:

1. Year organisation began offering the service: ____________________
2. Source of funding for this activity *(please put an X next to all that apply)*

____ a. Biomedical or other for-profit company

____ b. Government

____ c. Other - *please specify*:

1. Estimated annual budget for this activity (in US dollars): ________________
2. Estimated number of “services” produced per year (*please specify nature of service*): ____________________
3. Estimated time for production of a single “service”: ____________________

8. What formal relationships does your unit have with government, universities, and other national and international organisations?

8a. Please describe in detail any relationships that are particularly important or valuable:

1. What are the main strengths of how your organisation is organised?
2. What are the main weaknesses of how your organisation is organised?

**Why and how the organisation was established**

1. What background documents or resources were helpful in establishing your organisation?

12. Were examples from other countries helpful?

____ Yes → *Which examples?*

____ No

1. What other information would have been helpful in establishing the unit?
2. What advice would you give to others establishing a similar organisation?

**FOCUS**

1. Domains in which service can be provided *(please put an X next to all that apply)*

____ a. Primary healthcare

____ b. Secondary healthcare

____ c. Tertiary healthcare

____ d. Public health (*i.e., public health is the objective of the policy*)

____ e. Healthy public policy (*e.g.., economic, employment, housing or transport policies where the health of populations is a desired consequence of the policy but not necessarily a primary objective* )

1. Domains in which service can be provided *(please put an X next to all that apply)*

____ a. Characterizing health problems

____ b. Identifying potential solutions to health problems

____ c. Fitting potential solutions into health systems (*e.g., governance, financial and delivery arrangements*)

____ d. Bringing about change in health systems

1. Target users *(please put an X next to all that apply)*

____ a. Public policymakers in health departments

____ b. Public policymakers in other departments

____ c. Public policymakers in central agencies (*e.g., executive branch*)

____ d. Stakeholders

1. Involvement of target users in the services that you provide

____ a. Yes, by participation in working groups

____ b. Yes, by survey of views / preferences

____ c. Yes, by review of draft reports

____ d. No

1. Involvement of consumers (patients or representatives of the general public) in the services that you provide

____ a. Yes, by participation in working groups

____ b. Yes, by survey of views / preferences

____ c. Yes, by review of draft reports

____ d. No

**PEOPLE INVOLVED IN SERVICE DELIVERY**

1. Average number of staff involved in service delivery

____ a. < 0.5 full-time equivalents (FTE)

____ b. 0.5 – 1.9 FTE

____ c. > 2 FTE

1. Involvement of experts/stakeholders in supporting the service *(please put an X next to all that apply)*

Always involved Only if necessary

- 1. Informatics / library science ____ ____
  2. Clinical epidemiology ____ ____
  3. Biostatistics ____ ____
  4. Health economics ____ ____
  5. Other types of social scientists ____ ____
  6. Knowledge transfer / communication ____ ____
  7. Consumer ____ ____
  8. Other ____ ____ *please specify*:

**Methodology of response development**

1. Types of information employed *(please put an X next to all that apply)*

____ a. Systematic reviews

____ b. Economic evaluations

____ c. Decision analyses

____ d. Existing burden of disease/illness

____ e. Existing practice patterns

____ f. Existing guidelines (or HTAs)

____ g. Resource constraints

____ h. Commissioning of research

____ i. Other - *please specify*:

1. Explicit valuation process *(please put an X next to all that apply)*

____ a. Evidence is prioritized by its quality

____ b. Outcomes are prioritized by their importance to those affected

____ c. Groups are prioritized by their importance to achieving equity objectives

1. Methods used to formulate recommendations *(please put an X next to all that apply)*

____ a. Subjective review

____ b. Informal consensus

____ c. Formal consensus (*e.g., consensus conference, nominal group technique, Delphi technique*)

____ d. Graded according to the quality of the evidence and/or the strength of the recommendation (using an explicit rating scheme)

1. Explicit assessment *(please put an X next to all that apply)*

____ a. The quality of evidence

____ b. Trade-offs between benefits and harms

____ c. Costs

____ d. Equity

1. Review process *(please put an X next to all that apply)*

____ a. Policy validation (*e.g., pilot testing, trial implementation period*)

____ b. Comparison with input from other groups

____ c. Internal review

____ d. External review by experts

­­____ e. External review by target users

1. How does your organisation make decisions on which topics to work on?
2. What are the main strengths of the methods that you use?
3. What are the main weaknesses of the methods that you use?

**Products and implementation**

1. Versions produced *(please put an X next to all that apply)*

____ a. Full version with notes/references

____ b. Executive summary

____ c. Summary of take-home messages

____ d. Separate summaries/versions for different target users

____ e. Tools for application (*e.g., algorithms, flow charts*)

1. Does your unit send versions of its produces to the media as part of the dissemination/implementation strategy ?

____ a. Yes → *How?*

____ b. No → *Why not?*

1. Implementation strategies used *(please put an X next to all that apply)*

____ a. Mail or e-mail to target users

____ b. Produce a CD-ROM and distribute it to target users

____ c. Post to a website accessed by target users

____ d. Submit to a clearinghouse

____ e. Other - *please specify*:

1. Other implementation strategies used *(please put an X next to all that apply)*

____ a. Patient-mediated interventions - *please specify*:

____ b. Provider-mediated interventions (*e.g., audit and feedback*)

- *please specify*:

____ c. Organisational interventions (*e.g., change in setting of service delivery, coverage or reimbursement decision*) - *please specify*:

1. Strategies to develop the capacity of target users to acquire, assess and use research *(please put an X next to all that apply)*

____ a. Organize training workshops for target users

____ b. Participate in training workshops for target users

____ c. Develop a resource document for target users

____ d. Other – *please specify*:

1. Involvement of target users in implementation

____ a. Yes, by participation in implementation group

____ b. Yes, by survey of views / preferences

____ c. Yes, by review of draft implementation strategy

____ d. No

1. What are the main strengths of the outputs of your organisation?
2. What are the main weaknesses of the outputs of your organisation?

**EVALUATION AND UPDATE PROCEDURES**

1. Does you organisation collect data systematically about uptake?

____ a. Yes

____ b. No

1. Does your organisation systematically evaluate usefulness or impact in other ways?

____ a. Yes - *please specify*:

____ b. No

1. Does your organisation update its products?

____ a. Update regularly

____ b. Update irregularly

____ c. Do not update

**Additional questions**

1. Other types of support for the use of research evidence

____ a. Produce clinical practice guidelines

____ b. Produce HTAs

____ c. Other - *please specify*:

____ d. No

1. Who are the strongest advocates of your organisation and why?
2. Who are the strongest critics of your organisation and why?
3. Please add any other comments you have about the strengths of your organisation with respect to how you support the use of research evidence in developing clinical practice guidelines or health technology assessments, including:

- aspects of how you are organised or the methods that you use that you have found particularly useful
- innovations in how you are organised or the methods that you use,
- examples of successes that you have had

1. Please also add comments about any additional information that this project might provide that you would like to have or that you believe would be helpful to other organisations like yours.
2. What is your view about the current role the WHO, and other international organisations play in developing guidelines, recommendations, and helping policymakers to access and use research evidence?
3. What role do you think the WHO and other international organisations should play in developing guidelines, recommendations and helping policymakers to access and use research evidence?
4. Finally, are there other organisations like yours that you would suggest that we should include in our review?

Please feel free to describe some examples of the output of your unit:

**Thank you for participating in this survey!**

ID # ______
