# Supplementary material for: Evidence-informed health policy 2 – Survey of organizations that support the use of research evidence
Source: Implement Sci. 2008 Dec 17;3:54. doi: 10.1186/1748-5908-3-54 (PMC2646748; doi:10.1186/1748-5908-3-54)
Supplement: Additional file 3 — Qualitative data from the survey of organizations that support the use of research evidence. [file 1748-5908-3-54-S3.doc]

**Evidence-informed health policy: 2. Survey of organizations that support the use of research evidence**

**[Excerpt – Qualitative data from the survey of organizations that support the use of research evidence]**

John N. Lavis^1^*, Elizabeth J. Paulsen^2^, Andrew D. Oxman^2^, Ray Moynihan^3^

*Corresponding author

^1^Centre for Health Economics and Policy Analysis, Department of Clinical Epidemiology and Biostatistics, and Department of Political Science, McMaster University, 1200 Main St. West, HSC-2D3, Hamilton, ON, Canada L8N 3Z5

^2^Norwegian Knowledge Centre for the Health Services, Pb. 7004, St. Olavs plass, Oslo, Norway N-0130

^3^School of Medicine and Public Health, Faculty of Health, The University of Newcastle, Medical Sciences Building – Level 6, Callaghan, NSW, Australia 2308

Email addresses:

JNL: lavisj@mcmaster.ca

EJP: elizabeth.paulsen@kunnskapssenteret.no

ADO: oxman@online.no

RM: ray.moynihan@newcastle.edu.au

**Qualitative results — Organizations producing CPGs and HTAs**

**Organization**

For organizations producing CPGs and HTAs, the most common formal relationships are with government and parastatal agencies, professional bodies, and universities. The relationship with a government’s health department is typically regarded as the most valuable; however, the reasons for this assessment can be quite diverse, including the health department’s roles as a funder or as a principal target audience. Close relations with professional bodies are also commonly seen as valuable, typically because of their credibility with another target audience – clinicians. These organizations also have formal relationships with many international networks, including the Guidelines International Network (GIN), Health Technology Assessment International (HTAi), and International Network of Agencies for Health Technology Assessment (INAHTA). Relationships with these networks can provide peer-to-peer information exchange. Relationships with industry or consumer groups are rarely cited as particularly important or valuable.

Independence is by far the most commonly cited strength in how these organizations are organized. Typical responses to the question about an organization’s main strength include: ‘financial and intellectual independence’ and ‘freedom from government and industry influence.’ A lack of resources, both financial and human, is the most commonly cited weakness in how these organizations are organized. Financial resources are often inadequate or time-limited. Human resource limitations typically involve a lack of qualified staff (*e.g.*, methodological experts) and volunteers (*e.g.*, clinical experts) without significant conflicts of interest. A lack of resources can add significantly to the length of time it takes to produce a guideline or HTA, with some organizations citing a perception that they are too slow to respond to health system challenges. Another, less commonly cited weakness was the lack of implementation strategies and evaluations.

**Establishment**

The question about what background documents or resources were helpful in establishing the organization was variously interpreted as pertaining to the documents that led to the creation of the organization, to the documents that informed their CPG- or HTA-development processes, and (as was the question’s intent) to the documents that informed how the organization was established. No documents were cited repeatedly as being particularly helpful in informing how the organization was established, although many organizations conducted a focused review of one particular organization that they then emulated or a broad review of a variety of organizational models.

Few individuals responded to the question about what other information would have been helpful in establishing the organization, and there was no clear pattern in the responses of those who did. Some organizations did note that information about other similar organizations’ practical experiences may have been helpful in establishing the organization (*e.g.*, how to nurture a policy climate that supports evidence-based CPGs and HTAs, operational procedures for producing CPGs and HTAs, and time and resource estimates for producing CPGs and HTAs).

The advice that was most commonly offered to others establishing a similar organization was to seek support from similar existing organizations or networks, whether through informal interactions, study tours, mentoring relationships, twinning, partnerships or network memberships. Other advice for those establishing new organizations ranged from being clear about what need the organization will fill given existing local, national, and international initiatives to ensuring independence (both in terms of the organization itself and the individuals working in it) and establishing good relationships with key target audiences from the beginning.

**Methods used in producing a product or delivering a service**

Organizations tend to make decisions about which CPGs or HTAs to produce on the basis of explicit criteria such as disease burden, national priority area, and utilization, cost, or safety profile (*e.g.*, prescription drugs or technologies used in high volumes, at high cost or with safety concerns); input or requests from health policymakers, insurers, health system managers, clinicians, experts, and other stakeholders (through surveys and both formal and informal consultations); or both. For example, a professional body that produces guidelines used the following explicit criteria:

- conditions for which the diagnosis and management of the disease could be significantly improved by a change in practice;
- a body of published evidence exists on which to base the guideline;
- topic has wide variability in practice;
- topic is controversial;
- topic includes interventions that potentially have high economic cost;
- topic is of interest and importance to public health; and
- range of topics reflect the constituency of the professional body and cross over its various disciplines.

**Some organizations have an explicit timetable for updating their CPGs and HTAs.**

An evidence-based approach is the most commonly cited strength of the methods used by these organizations. In describing an evidence-based approach, survey respondents use terms such as systematic, explicit, transparent, rigorous, and reproducible. For example, one individual cited ‘to use an explicit and systematic methodology that can be reproducible’ as the main strength of the methods they use. As another example, systematic reviews are cited as a strong protection against bias in the identification, selection, appraisal and synthesis of the research literature on which CPGs and HTAs are based. Involving stakeholders in the process also emerged as a frequently cited methodological strength, although the focus tended to be more on clinicians than on citizens or consumers. A much smaller number of organizations cited flexibility and grading the strength of recommendations as methodological strengths.

The most commonly cited weakness of the methods used by these organizations was their time-consuming and labour-intensive nature. This weakness was felt very acutely by many organizations because it left them vulnerable to assertions that they were slow and insufficiently responsive. Other cited weaknesses included the insufficient involvement of citizens/consumers and a lack of or low quality evidence on priority topics.

**Products**

The main strength of the organizations’ outputs was commonly seen as the brand recognition that was perceived to flow from the organizations’ evidence-based approach and, much less commonly, from their strict conflict of interest guidelines. This brand recognition was typically spoken of in terms of either credibility and trustworthiness or high standards and quality. Other less commonly cited strengths included outputs being available in many different formats (*e.g.*, print and web-based, short summaries and full reports, and outputs written at different language levels) and outputs being timely, relevant to local needs, and locally applicable.

The most commonly cited weaknesses of the organizations’ outputs are the lack of dissemination and implementation strategies for the outputs and the lack of monitoring and evaluation of impact. Other weaknesses include a lack of user-friendly products and a lack of timeliness. One respondent reported ‘[w]e cannot produce enough new evidence in a timely fashion.’ Another reported ‘[The] development process takes too long. Recommendations are in danger to be outdated by the time they are published.’

**Advocates and critics**

The organizations’ strongest advocates are the individuals, groups and organizations who have worked with the organizations (*e.g.*, funders, heads of professional bodies, panel members, and researchers) or who have benefited from their outputs (*e.g.*, health policymakers making technology funding decisions, health professionals making clinical decisions). While the reasons for their support vary, most advocates appear to value participating in the production of CPGs and HTAs or being able to draw on independent and timely high-quality and high-relevance CPGs and HTAs.

The most commonly cited critic of the organizations was the pharmaceutical industry and clinicians who are closely associated with them. More generally, a respondent from one organization summed up the different types of critics quite succinctly: ‘[Critics] fall into three groups: those who disagree with our conclusions; those who would like to have more rapid conclusions/recommendations from HTAs; and those who do not see the value of HTAs. These groups are not mutually exclusive!’ Several organizations proudly cited people within their own organization as their strongest critic. For example, a respondent from one organization said that ‘the strongest critics are often our own staff. We foster a culture of self-examination and critique.’

**Role of WHO and other international organizations and networks**

A question gauging views about WHO’s and other international organizations’ current role in developing guidelines and HTAs and helping policymakers to access and use research evidence did not reap a rich set of responses. Many organizations in high-income countries focused on the importance of international networks such as GIN, HTAi, and INAHTA, whereas many organizations in LMICs focused on WHO’s role more generally and not specifically in the domain of CPGs and HTAs.

A question about what role WHO and other international organizations should play produced a richer set of responses. The most commonly cited suggestions were that WHO and other international organizations and networks play a facilitating role in coordination efforts (in order to avoid duplication) and in local adaptation efforts (in order to enhance local applicability). Other suggestions included: providing an ‘inventory of evidence’ and disseminating it on a global scale; producing guidelines for guideline developers; and supporting networks that in turn support others working in this field. The following selected quotes illustrate some of the key suggestions.

‘I'd also like to see better international collaborations that allow all of us to complement each other rather than so often having the reality or perception that we're duplicating each other.’

‘WHO has a key role in influencing practice worldwide. It may not be the best placed organization for developing guidelines, but it could endorse guidelines developed by national programmes and/or encourage access [to] and use of evidence across countries. Many of the guidelines developed in ‘advanced’ countries would not be applicable to all countries. Therefore there is a need to adapt existing guidelines, or find ways for using evidence/teaching in less developed countries.’

‘….promote information sharing on specific guidelines; encourage work on guideline or TA [technology assessment] adaptation; encourage work on issues that may not be relevant to one single country but to a region; encourage networking, particularly to help eastern countries.’

WHO ‘can use its prestige to support right and systematic methods to develop guidelines.’

**Qualitative results – Government support units**

There was a great deal more heterogeneity in the responses from the GSUs than in the responses from the organizations producing CPGs and HTAs.

**Organization**

For GSUs, the most common formal relationships are with government, and the most valuable of these relationships typically with government departments of health. One respondent replied: ‘Arm[s] length relationship with the Ministry of Public Health is important, not too close to be dominated and [not] too distant to be irrelevant.’ Some units also have formal relationships with professional associations, non-governmental organizations, universities, and international agencies.

Working within national networks and, more generally, collaborating rather than competing with other bodies, are a commonly cited strength in how these units are organized. Other cited strengths include a multi-disciplinary approach, an ability to respond quickly to requests, and links with universities. A GSU in a middle-income country cited as a strength: ‘Focusing in capacity-strengthening through apprenticeship of young researchers in health systems and policy research, expose to real policy arena and real life policy analysis and makings.’ The cited weaknesses in how the units are organised are also quite diverse: small size, inadequate funding, lack of critical mass of researchers, and difficulties retaining staff, as well as poor communication with stakeholders. As an example of the latter, one respondent wrote: ‘We have not communicated very well with the public or clinicians about the methods we use and the rationale for decisions made.’

**Establishment**

The only theme emerging from among responses to the series of questions about why and how the unit was established is the advice that those establishing a similar organization attend to the need for secure funding. For example on respondent said: ‘Be clear about the vision, mission, and get the right people well balanced in terms of passion and evidence base - have a secured funding!’ Another said: ‘Try and secure financial independence via core funding as early in the process as possible.’ No themes emerged in response to the question about what background documents or resources were helpful in establishing the unit or the question about what other information would have been helpful in establishing the unit.

**Methods used in producing a product or delivering a service**

While units tend to describe themselves as being ‘demand-driven’ (with the demand coming directly from government or indirectly from industry in terms of the appearance of a new drug or technology on the market) and less commonly indicate that they work out a consensus about priority topics with stakeholders, they do not cite explicit criteria as the basis for their priority-setting. No themes emerged among the diverse set of strengths and weaknesses cited in reference to the methods used by the units. Examples of strengths include using evidence-based approaches, focusing on cost-effectiveness, credibility, and openness and transparency, while examples of weaknesses include not assessing local needs and opportunities (‘priorities are sometimes donor-driven and do not result from a systematic analysis of needs and opportunities’), not obtaining clarity in the research question, the time-consuming nature of the methods, few or weak data, and inconsistent involvement of consumers.

**Products**

No strengths or weaknesses of the units’ outputs were consistently identified by respondents from the units. The cited strengths included high quality, valid methods, scientifically strong, trusted, respected, unbiased (which was equated with a lack of association with drug companies), and having products published in peer reviewed journals. The cited weaknesses included having a small staff or low budget to produce them, being too technical at times, not being transparent or publicly available, taking long to be published, and diffusing only slowly without proactive dissemination and implementation strategies. Several units cited poor dissemination and implementation per se.

**Advocates and critics**

The most commonly cited advocates are government health departments, with others including academics, stakeholders generally, and general practitioners and civil society activists in particular. A mix of critics was identified, including the pharmaceutical industry, some politicians, and some clinicians.

**Role of WHO and other international organizations and networks**

As with units that produce CPGs and HTAs, a question gauging views about WHO’s and other international organizations’ current role in developing recommendations and helping policymakers to access and use research evidence did not reap a rich set of responses among GSUs. A question about what role WHO and other international agencies should play produced a richer set of responses. The most commonly cited suggestion was that WHO should play a role in helping to adapt global evidence to local contexts or at least in supporting such processes. For example, one respondent suggested that WHO should take ‘a more proactive role in helping countries adapt existing guidelines to local and regional conditions.’
